# Supplementary material for: An Exploratory Study on the Physical Activity Health Paradox—Musculoskeletal Pain and Cardiovascular Load during Work and Leisure in Construction and Healthcare Workers
Source: Int J Environ Res Public Health. 2022 Feb 26;19(5):2751. doi: 10.3390/ijerph19052751 (PMC8910306; doi:10.3390/ijerph19052751)
Supplement: Supplementary file 1 [file ijerph-19-02751-s001.zip › ijerph-1549288-supplementary.pdf]

International Journal of
Environmental Research and Public Health

MDPI

Supplementary File

Table S1. Models describing the association between time spent ≥20 %HRR and overall musculosketal pain (scale 0-27); stratified by VO_2max, BMI, control of work pacing, and occupational sector. Associations with p<0.05 are marked in bold.

		$VO_2max < 34.8$ n = 36			$VO_2max \geq 34.8$ n = 36	
**20% HRR**	**mean**	**β**	**(95% CI)**	**mean**	**β**	**(95% CI)**
Intercept		3.60	(-0.89 – 8.04)		**3.91**	**(1.42 – 6.41)**
Time		0.23	(-0.11 – 0.58)		-0.24	(-0.52 – 0.04)
Awake/Sleep (*ilr*1)	-0.60	-1.98	(-9.54 – 5.51)	-0.54	-1.53	(-5.65 – 2.59)
Work/Leisure (*ilr*2)	-0.37	-3.77	(-9.13 – 1.55)	-0.41	-0.16	(-1.96 – 1.65)
≥20 %HRR Work/<20 %HRR Work (*ilr*3)	0.91	0.19	(-2.87 – 3.25)	0.66	0.45	(-0.96 – 1.87)
≥20% HRR Leisure/<20 %HRR Leisure (*ilr*4)	0.11	-0.81	(-3.24 – 1.58)	0.07	**-2.54**	**(-4.83 – -0.27)**

			BMI[¥]<25 n = 35			BMI[¥] ≥25 n = 37
**20% HRR**	**mean**	**β**	**(95% CI)**	**mean**	**β**	**(95% CI)**
Intercept		**3.36**	**(0.48 – 6.23)**		**4.48**	**(0.68 – 8.27)**
Time		0.07	(-0.24 – 0.37)		-0.05	(-0.38 – 0.27)
Awake/Sleep (*ilr*1)	-0.56	-1.14	(-5.59 – 3.28)	-0.57	-1.34	(-7.66 – 4.98)
Work/Leisure (*ilr*2)	-0.51	-0.85	(-3.58 – 1.88)	-0.29	-1.39	(-3.99 – 1.22)
≥20 %HRR Work/<20 %HRR Work (*ilr*3)	0.79	0.16	(-1.54 – 1.86)	0.77	1.58	(-0.39 – 3.58)
≥20% HRR Leisure/<20 %HRR Leisure (*ilr*4)	0.06	**-2.85**	**(-5.40 – -0.33)**	0.12	-1.39	(-3.76 – 0.97)

			Control of work pacing <2.75 n = 32			Control of work pacing ≥2.75 n = 38
**20% HRR**	**mean**	**β**	**(95% CI)**	**mean**	**β**	**(95% CI)**
Intercept		4.77	(-0.44 – 9.97)		**3.35**	**(0.43 – 6.28)**
Time		-0.14	(-0.45 – 0.16)		0.23	(-0.08 – 0.54)
Awake/Sleep (*ilr*1)	-0.61	-2.67	(-11.33 – 6.01)	-0.53	-0.69	(-5.05 – 3.66)
Work/Leisure (*ilr*2)	-0.48	-1.40	(-4.81 – 2.01)	-0.32	-0.94	(-3.99 – 2.12)
≥20 %HRR Work/<20 %HRR Work (*ilr*3)	1.09	0.13	(-2.40 – 2.68)	0.49	1.10	(-0.67 – 2.87)
≥20% HRR Leisure/<20 %HRR Leisure (*ilr*4)	0.15	-3.62	(-7.67 – 0.40)	0.06	-1.08	(-3.02 – 0.85)

			Construction n = 35			Healthcare n = 37
**20% HRR**	**mean**	**β**	**(95% CI)**	**mean**	**β**	**(95% CI)**
Intercept		**3.56**	**(0.65 – 6.47)**		**4.66**	**(0.63 – 8.69)**
Time		0.07	(-0.22 – 0.37)		-0.07	(-0.42 – 0.27)
Awake/Sleep (*ilr*1)	0.54	-0.76	(-6.11 – 4.58)	-0.59	-0.04	(-6.25 – 6.19)
Work/Leisure (*ilr*2)	-0.30	0.90	(-1.60 – 3.41)	-0.48	-2.20	(-5.49 – 1.09)
≥20 %HRR Work/<20 %HRR Work (*ilr*3)	0.87	1.20	(-0.88 – 3.27)	0.69	1.11	(-0.72 – 2.96)
≥20% HRR Leisure/<20 %HRR Leisure (*ilr*4)	0.22	-0.86	(-3.17 – 1.43)	-0.04	-1.52	(-4.47 – 1.40)

¥ BMI (Body mass index)

Table S2. Models describing the association between time spent ≥30 %HRR and overall musculoskeletal pain (scale 0-27); stratified by VO_2max, BMI, control of work pacing, and occupational sector. Associations with p<0.05 are marked in bold.

			VO_2max < 34.8 n = 36			VO_2max ≥ 34.8 n = 36
**30% HRR**	**mean**	**β**	**(95% CI)**	**mean**	**β**	**(95% CI)**
Intercept		5.44	(-0.71 – 11.50)		1.45	(-2.11 – 4.99)
Time		0.23	(-0.11 – 0.58)		-0.24	(-0.52 – 0.04)
Awake/Sleep (*ilr1*)	-0.65	-0.89	(-10.35 – 8.44)	-0.64	-1.57	(-5.74 – 2.61)
Work/Leisure (*ilr2*)	-0.10	-3.32	(-9.83 – 3.14)	-0.15	-0.38	(-2.11 – 1.36)
≥30 %HRR Work/<30 %HRR Work (*ilr3)*	-0.65	2.06	(-1.51 – 5.68)	-0.72	0.67	(-0.85 – 2.19)
≥30% HRR Leisure/<30 %HRR Leisure (*ilr4*)	-1.01	-1.28	(-4.75 – 2.17)	-1.12	-2.57	(-5.19 – 0.03)

			BMI[¥] <25 n = 35			BMI[¥] ≥25 n = 37
**30% HRR**	**mean**	**β**	**(95% CI)**	**mean**	**β**	**(95% CI)**
Intercept		2.12	(-1.85 – 6.06)		**7.50**	**(3.30 – 11.70)**
Time		0.07	(-0.23 – 0.38)		-0.06	(-0.38 – 0.27)
Awake/Sleep (*ilr1*)	-0.64	-1.19	(-5.97 – 3.57)	-0.66	0.33	(-7.19 – 7.85)
Work/Leisure (*ilr2*)	-0.24	-0.49	(-3.12 – 2.14)	-0.02	-1.07	(-3.95 – 1.81)
≥30 %HRR Work/<30 %HRR Work (*ilr3)*	-0.63	0.45	(-1.39 – 2.30)	-0.74	1.68	(-0.80 – 4.18)
≥30% HRR Leisure/<30 %HRR Leisure (*ilr4*)	-1.00	-1.68	(-4.60 – 1.22)	-1.12	-0.59	(-3.39 – 2.19)

			Control of work pacing <2.75 n = 32			Control of work pacing ≥2.75 n = 38
**30% HRR**	**mean**	**β**	**(95% CI)**	**mean**	**β**	**(95% CI)**
Intercept		**7.29**	**(1.02 – 13.53)**		**3.99**	**(0.46 – 7.48)**
Time		-0.14	(-0.45 – 0.16)		0.22	(-0.09 – 0.53)
Awake/Sleep (*ilr1*)	-0.60	0.14	(-9.58 – 9.83)	-0.70	-0.51	(-5.43 – 4.39)
Work/Leisure (*ilr2*)	-0.07	-0.12	(-3.45 – 3.20)	-0.20	-0.77	(-3.89 – 2.35)
≥30 %HRR Work/<30 %HRR Work (*ilr3)*	-0.39	-0.19	(-3.07 – 2.71)	-0.98	1.31	(-0.96 – 3.58)
≥30% HRR Leisure/<30 %HRR Leisure (*ilr4*)	-0.98	0.63	(-4.60 – 5.83)	-1.13	-1.13	(-3.35 – 1.07)

			Construction n = 35			Healthcare n = 37
**30% HRR**	**mean**	**β**	**(95% CI)**	**mean**	**β**	**(95% CI)**
Intercept		**4.65**	**(1.02 – 8.26)**		**6.15**	**(1.45 – 10.81)**
Time		0.07	(-0.22 – 0.37)		-0.07	(-0.41 – 0.27)
Awake/Sleep (*ilr1*)	-0.58	0.09	(-5.16 – 5.32)	-0.71	-1.21	(-8.75 – 6.31)
Work/Leisure (*ilr2*)	0.01	0.82	(-1.59 – 3.25)	-0.25	-2.57	(-6.55 – 1.39)
≥30 %HRR Work/<30 %HRR Work (*ilr3)*	-0.51	0.64	(-1.11 – 2.39)	-0.85	2.56	(-0.57 – 5.74)
≥30% HRR Leisure/<30 %HRR Leisure (*ilr4*)	-1.04	-0.27	(-2.63 – 2.09)	-1.09	-1.01	(-4.68 – 2.61)

¥ BMI (Body mass index)

Table S3. Models describing the association between time spent ≥ 40 %HRR and overall musculoskeletal pain (scale 0-27); stratified by VO_2max, BMI, control of work pacing, and sector. Associations with p<0.05 are marked in bold.

			**VO_2max < 34.8 n = 36**			**VO_2max ≥ 34.8 n = 36**
**40% HRR**	**mean**	**β**	**(95% CI)**	**mean**	**β**	**(95% CI)**
Intercept		**7.49**	**(2.87 – 12.08)**		0.60	(-4.41 – 5.57)
Time		0.24	(-0.11 – 0.58)		-0.23	(-0.52 – 0.04)
Awake/Sleep (*ilr1*)	-1.19	-0.47	(-9.64 – 8.59)	-1.27	-1.92	(-6.42 – 2.58)
Work/Leisure (*ilr2*)	-0.29	-2.15	(-8.77 – 4.45)	-0.13	-0.56	(-2.44 – 1.31)
≥40 %HRR Work/<40 %HRR Work (*ilr3*)	-2.16	1.90	(-3.99 – 7.85)	-2.20	0.83	(-1.26 – 2.90)
≥40% HRR Leisure/<40 %HRR Leisure (*ilr4*)	-2.01	-0.86	(-4.97 – 3.25)	-2.36	-1.54	(-3.90 – 0.81)

			**BMI[¥] <25 n = 35**			**BMI[¥] ≥25 n = 37**
**40% HRR**	**mean**	**β**	**(95% CI)**	**mean**	**β**	**(95% CI)**
Intercept		1.67	(-3.01 – 6.28)		**9.57**	**(5.14 – 13.99)**
Time		0.08	(-0.23 – 0.39)		-0.05	(-0.38 – 0.27)
Awake/Sleep (*ilr1*)	-1.19	-1.69	(-6.62 – 3.21)	-1.26	0.21	(-8.07 – 8.53)
Work/Leisure (*ilr2*)	-0.29	-0.30	(-3.15 – 2.54)	-0.13	-1.20	(-4.34 – 1.94)
≥40 %HRR Work/<40 %HRR Work (*ilr3*)	-2.08	0.64	(-2.03 – 3.32)	-2.27	1.77	(-2.04 – 5.57)
≥40% HRR Leisure/<40 %HRR Leisure (*ilr4*)	-2.12	-0.93	(-3.57 – 1.69)	-2.25	-0.58	(-3.77 – 2.61)

			**Control of work pacing <2.75 n = 32**			**Control of work pacing ≥2.75 n = 38**
**40% HRR**	**mean**	**β**	**(95% CI)**	**mean**	**β**	**(95% CI)**
Intercept		**8.70**	**(1.65 – 15.74)**		**4.15**	**(0.25 – 8.00)**
Time		-0.14	(-0.45 – 0.17)		0.22	(-0.09 – 0.53)
Awake/Sleep (*ilr1*)	-1.11	-0.70	(-10.54 – 9.11)	-1.34	-0.84	(-5.89 – 4.18)
Work/Leisure (*ilr2*)	0.02	0.43	(-2.94 – 3.79)	-0.44	-0.61	(-3.78 – 2.57)
≥40 %HRR Work/<40 %HRR Work (*ilr3*)	-1.80	-0.40	(-4.44 – 3.66)	-2.56	1.31	(-1.66 – 4.29)
≥40% HRR Leisure/<40 %HRR Leisure (*ilr4*)	-2.11	1.67	(-2.88 – 6.22)	-2.24	-1.03	(-3.35 – 1.30)

			**Construction n = 35**			**Healthcare n = 37**
**40% HRR**	**mean**	**β**	**(95% CI)**	**mean**	**β**	**(95% CI)**
Intercept		**4.43**	**(0.09 – 8.76)**		**8.80**	**(3.92 – 13.66)**
Time		0.07	(-0.22 – 0.37)		-0.07	(-0.41 – 0.27)
Awake/Sleep (*ilr1*)	-1.15	-0.33	(-6.10 – 5.42)	-1.30	-0.80	(-8.47 – 6.86)
Work/Leisure (*ilr2*)	0.06	0.54	(-2.05 – 3.13)	-0.46	-2.40	(-6.81 – 2.01)
≥40 %HRR Work/<40 %HRR Work (*ilr3*)	-1.98	0.31	(-2.30 – 2.92)	-2.37	2.95	(-1.63 – 7.57)
≥40% HRR Leisure/<40 %HRR Leisure (*ilr4*)	-2.28	-0.14	(-2.58 – 2.31)	-2.09	-1.25	(-5.04 – 2.50)

[¥] *BMI (Body mass index)*
